# The role of inflammation in secondary cardiac dysfunction following trauma

**DOI:** 10.1186/1757-7241-22-S1-P12

**Published:** 2014-07-07

**Authors:** Nick M Wilson, Henry D De’Ath, Joanna Manson

**Affiliations:** 1 Trauma Sciences, Queen Mary University, London, UK

## Background

Recent evidence supports the existence of a trauma induced secondary cardiac injury (TISCI) that is associated with poor clinical outcome [1]. Cardiac dysfunction has been demonstrated as early as 30mins after injury and is characterised by reduced contractility that is unresponsive to increased perfusion and preload [2].Inflammation is implicated as a key aetiological factor [1,3]. This review aims to evaluate current knowledge on cardiac inflammation after trauma and highlight mechanisms for future study.

## Method

A literature search for studies pertaining to cardiac inflammation after mechanical trauma (T), haemorrhagic shock (HS) or both combined (THS) was performed.

## Results

The search identified 1 clinical and 25 pre-clinical studies for inclusion.

Following T/HS and THS there is an increase in cardiomyocyte interleukin-6 (IL-6), tumour necrosis factor-alpha (TNF-a) and nuclear factor Kappa-B (NFKB). A rise in intracellular adhesion molecule-1 (ICAM-1) expression in cardiac tissue has also been observed. Neutrophil infiltration of cardiac tissue has been observed 6h after injury. Following T/HS and THS cardiac inducible nitric oxide synthase (iNOS) and nicotinamide adenine dinucleotide phosphate-oxidase (NADPH) levels increases suggested to be associated with a rise in reactive oxygen and nitrogen species. In addition, significant levels of apoptosis have been observed peaking at 12h following injury.

Pharmacological agents including oestrogen and insulin have been shown to reduce cardiac inflammation and prevent contractile dysfunction. The precise mechanism of this cardio-protection is yet unknown but involvement in preventing oxidative and nitrative stress is apparent.

## Conclusion

Cytokine production, cell activation, neutrophil infiltration and production of reactive oxygen and nitrogen species have been observed in cardiac tissues after trauma with corresponding reduction in intrinsic myocardial contractility. This review has highlighted some mechanisms which require further study if we are to understand the complex clinical sequelae of trauma and possibly develop novel therapeutics.

## References

[B1] De'Ath HD Manson J Davenport R Glasgow S Renfrew I Davies LC Trauma-induced secondary cardiac injury is associated with hyperacute elevations in inflammatory cytokines Shock 2013 39 5 415 20 10.1097/SHK.0b013e31828ded41 23459112

[B2] Shahani R Klein LV Marshall JG Nicholson S Rubin BB Walker PM Hemorrhage-induced alpha-adrenergic signaling results in myocardial TNF-alpha expression and contractile dysfunction Am J Physiol Heart Circ Physiol 2001 281 1 H84 92 11406472 10.1152/ajpheart.2001.281.1.H84

[B3] Kher A Wang M Tsai BM Pitcher JM Greenbaum ES Nagy RD Sex differences in the myocardial inflammatory response to acute injury Shock 2005 23 1 1 10 10.1097/01.shk.0000148055.12387.15 15614124

